# Chorda tympani injury during endoscopic versus microscopic stapes surgery: a randomized controlled clinical trial

**DOI:** 10.1007/s00405-022-07550-0

**Published:** 2022-07-24

**Authors:** Waleed Moneir, Hesham Mohamed Eladl, Moataz Mohammed El-Okda, Hisham Atef Ebada

**Affiliations:** Department of Otorhinolaryngology, Mansoura University, Mansoura, 35511 Egypt

**Keywords:** Chorda tympani, Stapedotomy, Endoscopic, Microscopic

## Abstract

**Objectives:**

The aim of this study was to compare the incidence of chorda tympani nerve (CTN) injury between endoscopic and microscopic stapes surgery.

**Methods:**

This randomized controlled clinical trial included 88 patients who were randomly divided into two groups: endoscopic stapedotomy group (*n* = 44) and microscopic stapedotomy group (*n* = 44). The incidence of chorda tympani nerve (CTN) injury after surgery was determined by both subjective taste testing and chemical taste tests, before and after surgery. The results were compared between the two groups.

**Results:**

The total number of patients who were identified as having CTN affection (based on the chemical testing) was 16 out of 88 (18.2%). The incidence was significantly lower in the endoscopic group (*n* = 2) than the microscopic group (*n* = 14) (*p* = 0.019).

**Conclusion:**

Altered taste as a result of iatrogenic CTN injury can affect the patients’ quality of life. Endoscopic ear surgery offers better visualization, less need for extensive manipulation of the chorda tympani, and consequently decreased incidence of CTN injury.

## Introduction

The chorda tympani nerve (CTN) is a branch of the seventh cranial nerve in the middle ear. It has sensory fibers that supply taste by innervating the anterior two thirds of the tongue, as well as secretomotor fibers that innervate the submandibular and sublingual glands [1].

Due to its course inside the tympanic cavity and its relationship with the ossicular chain, the CTN is at risk for iatrogenic injury during middle ear surgery, with consequent transitory or permanent dysgeusia [2].

The CTN may be injured during several middle ear surgical procedures including tympanoplasty, mastoidectomy, meatoplasty and stapes surgery [3]. During stapes surgeries, manipulation of the CTN is often necessary to widen the surgical view, and to create a favorable working area on the stapes [4].

The most common symptom of the CTN injury is change in taste sensation [1, 3]. Taste impairment could affect patients’ quality of life to different degrees [5]. Some report negligible taste disturbances, whereas others state that the effects are detrimental [4, 6].

The recent increase in the use of endoscope in ear surgeries in the past two decades allowed for better visualization of the middle ear and ossicular chain, particularly when using angled scopes. Endoscopy has rendered extensive and rough manipulation of the CTN less necessary, thereby minimizing iatrogenic injury [1, 7, 8].

The aim of this study was to compare the incidence of CTN injury between endoscopic and microscopic stapes surgery.

## Patients and methods

This randomized controlled clinical trial was conducted in the Otorhinolaryngology Department, Faculty of Medicine, Mansoura University, over 2 years (June 2019–June 2021). The study was approved by the Mansura Faculty of Medicine Institutional Research Board (MFM-IRB: MS.19.11.907). An informed written consent was obtained from all patients involved in the study.

### Patients

All adult patients with the clinical diagnosis of otosclerosis, who were scheduled for stapedotomy during the 2 years of the study (*n* = 97), were eligible for inclusion. Five patients were excluded from the study due to previous middle ear surgery (four patients underwent previous stapedotomy, and one patient underwent previous tympanoplasty). Three patients were excluded as the intraoperative findings revealed other causes of conductive hearing loss (incudo-stapedial disarticulation in two patients and malleolus fixation in one). One patient was excluded due to pre-existing taste disorder. The remaining 88 patients were evaluated.

Patients of the study (*n* = 88) were randomly divided into two groups: endoscopic stapedotomy group (*n* = 44) and microscopic stapedotomy group (*n* = 44). Randomization was performed based on closed envelope method.

### Surgical procedure

In both groups, all surgeries were performed via a trans-canal approach under local anesthesia. All endoscopic surgeries were performed by the senior author (WM), while all microscopic surgeries were performed by the second author (HME). The local anesthetic solution (2% lidocaine 1:50,000 epinephrine) was injected into the tragus as well as four quadrant injection in the external auditory canal just lateral to the osseocartilaginous junction.

The tympano-meatal flap was raised down to the annulus and elevated from the annular sulcus. The middle ear was entered preferentially at the postero-inferior annulus to facilitate identification of the CTN and minimize its traction or avulsion. The tympanic membrane was then reflected anteriorly to the level of the malleus neck and lateral process. An ear curette was used to partially remove part of the scutum to allow sufficient access for the endoscope and instruments.

The mobility of the ossicular chain was tested to ensure the diagnosis of otosclerosis. A perforation then was made by a manual perforator (0.8 mm in diameter) in the footplate then insertion of the Teflon piston (0.6 mm in diameter) was done.

The stapedius tendon was then cut with a sickle knife, and both anterior and posterior crus of the stapes were divided with a micro scissor. The incudo-stapedial joint was then separated with a joint knife or small right-angled micro pick. Next, the stapes suprastructure was fractured and removed. The tympanomeatal flap was relocated and then hearing was tested.

### Gustatory testing

All patients were subjected for gustatory testing preoperatively, and 48 h after surgery to assess the function of the chorda tympani. Gustatory testing was done using a questionnaire and chemical testing.

The authors of this work adopted the questionnaire proposed by Clark and O'Malley [9] who formulated the questionnaire by communication with the Pennsylvania Smell and Taste Center and The Taste and Smell Clinic, Washington to improve validity. The questionnaire assessed taste on a six-level scale ranging from extremely reduced taste to strongly improved taste. The purpose of the questionnaire was to assess changes in subjective taste.

Regarding the chemical test, the authors of the current study followed the same principles adopted by Melis and Barbarossa [10] and Claerhout et al. [11]. Identification of representative solutions of the five primary taste qualities (sweet, sour, salty, bitter, and umami) was tested. Two concentrations for each stimulus were used: a low concentration just above the recognition threshold and a high concentration to be clearly supra-threshold. Distilled water was used as a solvent. Table 1 shows the different substances and concentrations used.

**Table 1:** The substances and concentrations used for different taste qualities

Taste quality	Substance	Low concentration (mmol/L)	High concentration (mmol/L)
Sweet	Sucrose	20	146
Slaty	NaCl	20	85
Sour	Citric acid	1.3	5.2
Bitter	Caffeine	1.5	6.7
Umami	Monosodium glutamate (MSG)	10	80

Strips soaked with the different solutions were wiped on the lateral side of the anterior two-thirds of the extended tongue, on the same side of the ear surgery. The patients were instructed to leave the tongue out of the mouth to avoid distributing the solution over the whole mouth with the tongue. They were also asked to rinse their mouths with water after each application. The test was repeated ten times (the high and low concentration of each of the five taste qualities). The patients were instructed to identify the taste quality from a list of five descriptors, i.e., sweet, sour, salty, bitter and umami (multiple forced-choice procedure) and then place a mark. Then the right answers will be added to give a total score from 10.

The taste tests were performed in a blinded fashion, and the results of the tests were documented, tabulated, and analyzed. The function of the CTN was considered affected when there was decrease in the postoperative score of 30% or more, compared to the preoperative score of the patient.

## Results

This study included 88 patients: 39 males (44.3%) and 49 females (55.68%). Age of the patients ranged from 18 to 60 years (mean 36). Forty-seven surgeries (53.4%) were performed on the right ear, while 41 surgeries (46.5%) were performed on the left ear.

Intraoperative CTN manipulation were reported and documented (Table 2). In the microscopic group, the nerve was gently manipulated in 40 patients and grossly injured in 4 patients; stretched in 3 and cut in 1. On the other hand, in the endoscopic group, the nerve was gently manipulated in 42 patients, and it was grossly injured (stretched) in 2 patients.

**Table 2:** Intraoperative manipulation of the chorda tympani in the study groups

Group	Not manipulated	Gently manipulated without gross injury	Grossly injured
Microscopic stapedectomy	0	40	4
Endoscopic stapedectomy	0	42	2

Regarding postoperative subjective taste perception, altered taste was significantly higher in the microscopic group, where it was reported in 8 patients (18.2%), compared to the endoscopic group where it was reported in 2 patients (4.5%) (*p* = 0.019). These symptoms were temporary, and all the patients were free from symptoms within 1–6 months (average: 2.5 months).

Regarding the chemical testing, the average preoperative scores in the microscopic group was 9.41 ± 0.79, while the average postoperative scores was significantly lower (7.32 ± 2.68), with a 22.2% decrease (*p* = 0.002). On the other hand, the percentage of decrease in the scores in the endoscopic group was 9.9%, as the average preoperative scores was 9.14 ± 1.04, and the average postoperative scores was 8.23 ± 2.11. The postoperative scores in the endoscopic group were higher than the microscopic group (Table 3).

**Table 3:** The average preoperative and postoperative chemical test scores in both groups

	Microscopic	Endoscopic
Average taste scores		
Pre	9.41 ± 0.79	9.14 ± 1.04
Post	7.32 ± 2.68	8.23 ± 2.11
Paired *t* test	*p* = 0.002*	*p* = 0.061
Percent of affection	22.2%	9.9%

In this study, the total number of patients who were identified as having CTN affection (based on the chemical testing) was 16 out of 88 (18.2%). The incidence was significantly lower in the endoscopic group (*n* = 2) than the microscopic group (*n* = 14) (*p* = 0.019) (Table 4).

**Table 4:** The incidence of chorda tympani affection in both groups

	Microscopic stapedectomy, *n* = 44	Endoscopic stapedectomy, *n* = 44	Test of significance
Affected	14 (31.8%)	2 (4.5%)	*χ*^2^ = 5.5 *p* = 0.019
Not affected	30 (68.2%)	42 (95.5%)	

*χ*^2^ = Chi-Square test

## Discussion

The exposed course of the CTN in the middle ear makes it prone to damage not only by disease processes, but also by iatrogenic injury during middle ear surgery [1]. Mechanisms by which the CTN may be injured during surgery include transection (accidental or deliberate), stretching, ischemia, thermal injury, excessive handling, and desiccation.

Stretching occurs frequently during tympano-meatal flap elevation during tympanoplasty, or during exposure of the ossicular chain during stapedectomy or ossiculoplasty [12]. In the current study, the CTN was grossly damaged in six cases (stretched in five and cut in one). The gross damage was higher in the microscopic surgeries (*n* = 4) than in the endoscopic surgeries (*n* = 2).

The use of the endoscope during otologic surgery is associated with lower incidence of stretching and damage of the CTN, compared to microscopic surgery. The wide view of the endoscope allows identification of the nerve and its gentle manipulation. Lade et al. [7] and Iynen and Dundar [1] concluded that the risk of CTN stretching is lower in endoscopic surgery, due good exposure of the nerve without the need of curettage of the posterosuperior wall of the external auditory canal in most cases managed by endoscopic tympanoplasty.

Iynen and Dundar [1] reported that CTN was manipulated in 100% of patients undergoing microscopic surgery, and in only 32% of patients undergoing endoscopic surgery. In a previous study done by Moneir et al. [8] it was found that after elevation of the tympanomeatal flap, the incudo-stapedial joint, stapes suprastructure and stapedial tendon could be easily visualized by just advancing the endoscope toward the middle ear while tilting it. Minimal curettage of the posterosuperior meatal wall was needed for appropriate fenestration of the footplate and insertion of the prosthesis. In the current study, the use of endoscope offered less curettage and more gentle manipulation of the CTN, with better preservation of its function.

There is debate regarding heat issues in endoscopic ear surgery, with a limited body of work documenting potential negative impacts of middle-ear heat exposure from endoscopes [13]. Nogueira et al. [14] stated that the middle ear cavity is small and to have a great view the surgeon needs no more than 40% of light power. Moreover, Mitchell and Coulson [13] recommended that continuous exposure of the endoscope to the middle ear, in the absence of any cooling mechanism, should be limited and, ideally, should not exceed 5 min. Regular breaks of longer than 88 s, or regular application of suction or irrigation should be considered. In the current study, the light power was adjusted to 40% with regular breaks and irrigations to eliminate the possibility of thermal injury from the endoscope.

Molinari et al. [15] performed a cadaveric study to demonstrate the endoscopic anatomy and anatomical variations of the CTN. They concluded that the degree of overhanging of the scutum and the chordal eminence (whether absent or shallow versus intermediate or prominent) may influence the visibility of the oval window region during stapes surgery. During curetting or drilling the scutum and chordal eminence, the chorda tympani is at risk of being stretched or inadvertently cut. In the current study, the degree of overhanging of the scutum and the chorda eminence was not found to affect the incidence of CTN injury.

The most common reported symptom of CTN injury is altered taste sensation [3]. Altered taste perception after CTN injury has been reported in 15–22% of patients [3]. In the current study, 10/88 patients (11.4%) reported altered taste sensation. Interestingly, the incidence of postoperative altered taste sensation was significantly higher in patients who undergone microscopic surgery (8/44; 18.2%), than those who underwent endoscopic surgeries (2/44; 4.5%) (*p* = 0.019). Endoscopic surgery provides better visualization, and consequently less vigorous manipulations of the structures including the CTN, with less intraoperative and postoperative complications.

Subjective changes in somatosensory function of the tongue (tongue numbness) was also reported after CTN injury. Sakaguchi et al. [16] reported that the incidence of tongue numbness was 31.5%, after CTN injury. Similarly, Sakagami et al. [17] reported that 37.1% of patients with chronic otitis media complained of gustatory and trigeminal symptoms after surgery. Attenuation of the trigeminal sensation on the tongue after CTN injury was explained by some investigators. They suggested that part of lingual general sensation was possibly under control of the CTN [18, 19]. In contrast to these reports, none of the patients in our study complained of tongue numbness after surgery.

Postoperative subjective taste changes are usually not persistent. In the current study all patients recovered this symptom within 1–6 months (average: 2.5 months). Similarly, Gopalan et al. [6] reported that symptoms of CTN injury improved within 4–8 months. Michael and Raut [4] reported that a period of 12 months was required for full recovery of symptoms in his study.

Subjective taste recovery can be explained by multiple mechanisms. First, CTN recovery may occur after its injury. Other mechanisms include compensation of other nerves and subjective adjustment. The afferent inputs of the seventh and ninth cranial nerves have been shown to inhibit each other centrally. Consequently, a loss of CTN function can lead to an increase of taste sensitivity by the glossopharyngeal nerve [5]. Cross-innervation and compensation of the contralateral CTN and ipsilateral glossopharyngeal nerve have also been suggested [20]. McManus et al. [3] suggested that central adaptation to taste dysfunction occurs over time; however, it is uncertain whether the patient stops noticing the deficit or just stops complaining about it.

In the present study, the chemical taste tests showed postoperative reduction in the taste scores in 16/88 patients (18.2%). McManus et al. [21] reported that reduction of subjective taste occurred in 15–22% of patients after middle ear surgery. Clark and O'Malley [9] reported a rate of 30–38%. The incidence of reduction of postoperative taste scores was higher in the microscopic surgeries than in the endoscopic surgeries. The difference was statistically significant (*p* = 0.019). These results emphasize the value of the endoscopic surgery in preserving the function of the CTN.

Interestingly, among the 16 patients who showed reduction in the test scores postoperatively, only 10 patients (62.5%) complained of altered taste sensation. This poor correlation between patients’ complaints and test scores may be explained by the compensation of taste perception by the contralateral CTN or the glossopharyngeal nerve. On the other hand, correlation between chemical taste testing and subjective taste changes has been studied by Claerhout et al. [11]. They found significant correlation immediately after surgery, and loss of this correlation on the long term.

Endoscopic ear surgery has rendered CTN manipulation less necessary and has decreased the incidence of CTN injury [7]. The structural preservation rate of the CTN during endoscopic ear surgery was reported to be 100% by Molinari et al. [2], 100% by Migirov and Wolf [22], and 98,1% by Marchioni et al. [23]. CTN injury is not life-threatening and usually not permanent, however it affects patients’ quality of life, and therefore every effort should be made to prevent its injury. The risk of CTN injury during middle ear surgery can be minimized if an endoscopic technique is used.

## Conclusion

Altered taste as a result of iatrogenic CTN injury can affect the patients’ quality of life. Endoscopic ear surgery offers better visualization, less need for extensive manipulation of the chorda tympani, and consequently decreased incidence of CTN injury.
